# Effectiveness of mangrove sword bean food bar addressed to older people of landslide disaster victims

**DOI:** 10.3389/fnut.2024.1291580

**Published:** 2024-04-08

**Authors:** Fatmah Fatmah

**Affiliations:** Disaster Management Study Program, School of Environmental Science Universitas Indonesia, Jakarta, Indonesia

**Keywords:** older people, landslide, disaster, weight, mangrove-sword bean food bar

## Abstract

**Background:**

Older people require extra attention due to their reduced ability to prepare for disasters, as they adequately possess distinct needs. These groups necessitate uncomplicated, readily consumable, and palatable food options that fulfill their micronutrient needs. The objective of this research was to assess the effects of a snack bar enriched with *api-api* mangrove (*Avicennia marina*) and sword bean (*Canavalia ensiformis*) on the body weight and Body Mass Index (BMI) of older people individuals afflicted by a landslide event.

**Methods:**

A non-randomized pre-post-intervention study was undertaken, involving 31 senior participants. The intervention group consisted of 15 seniors who were provided with a mangrove sword bean snack bar, while the control group comprised 16 seniors who received a sword bean food bar during 15 days. All study participants received education on maintaining a balanced diet for older people individuals. The data analysis involved using univariate and bivariate analyses, explicitly applying the independent *t*-test and dependent *t*-test.

**Results:**

In the hedonic evaluation, the mangrove sword bean food bar had superior average attributes in terms of scent, flavor, texture, and color compared to the sword bean food bar. The consumption of snack bars made from mangrove sword beans resulted in a significant rise in weight (0.2 kg), energy intake (240.8 kcal), protein content (5.8 g), carbohydrate content (40.06 g), and fat content (4.4 g). Carbohydrate can significantly increase weight in the treatment subjects. Furthermore, the provision of comprehensive nutrition education has the potential to enhance the post-study knowledge score, as seen by the observed increase of 40.6. A significant disparity was observed between the mean carbohydrate consumption and understanding of balanced nutrition among the intervention and control groups.

**Conclusion:**

*Api-api* mangrove sword bean snack bars have been identified as a viable and efficient substitute for emergency food provisions, particularly in disaster-stricken communities. These food bars have demonstrated a significant capacity to contribute to the weight gain of individuals within such groups, thus addressing the nutritional needs of impacted populations in the aftermath of natural calamities. Subsequent investigations may include employing pregnant women as participants to explore the issue above.

**Clinical Trial Registration:**

Clinicaltrials.gov, identifier: NCT05897892.

## Introduction

Post-hydrometeorological disasters present a substantial problem in meeting the dietary requirements of vulnerable groups, particularly older people. To solve this matter, it is recommended that Complete Emergency Food Products (EFP) be made readily accessible for immediate consumption as the principal means of sustenance for 15 days, starting from the moment of evacuation (1, 2). In Indonesia, emergency food formulations include food bars created from a combination of brand and maize flours (3), cookies prepared with Moringa leaf flour (4), cookies made from sweet potato flour, banana flour, and mung bean flour (5), as well as a snack bar consisting of soybean, broccoli, and mangrove fruit (6).

The exploration of utilizing *api-api* mangrove (*Avicennia marina*) in producing food bars has yet to be previously investigated, despite its potential as a suitable alternative to rice and wheat flour. Based on a study conducted by researchers (7), it has been shown that the *api-api* mangrove demonstrates a greater energy density in comparison to rice and corn. The api-api mangrove is more energy-dense (371 kcal) than wheat flour (365 kcal) (7), rice (360 kcal), and corn (307 kcal) (8, 9). Api-api mangrove is commonly found in coastal tropical countries, including Indonesia. It is a plant species that is widely distributed throughout Indonesia and is abundant from Sabang to Merauke. *Avicennia marina* is a type of mangrove that can thrive in environments with minimal light and temperature, in brackish areas with high salinity levels, and is commonly found in coastal and mangrove habitats. They can also be found in freshwater swamps and muddy coastal areas with high salt content (10, 11).

Food bars produced from this specific wheat variety require additional lipids, carbohydrates, proteins, zinc, and vitamin C. *Canavalia ensiformis* (sword bean) flour has been selected to enhance geriatric nutrition. The snack bar has characteristics that render it a viable choice for dissemination among individuals affected by calamities. The compact and sturdy design facilitates convenient portability and manipulation. Furthermore, the creation of this product can be customized to include a wide variety of locally sourced food items, hence increasing its versatility and accessibility in various geographical areas. Furthermore, this substance can be ingested orally without needing any pre-treatment, and it exhibits a significant quantity of carbs and proteins. Similarly, when comparing food bars to cookies or pastries, it is essential to note that they can withstand higher amounts of pressure due to their partially dry composition (8). A comparative analysis was conducted in 2020 to assess the suitability of lindur fruit (*Bruguiera gymnorrhiza*) flour, broccoli flour, and soy flour as potential ingredients in a bar snack. During 15 days, a total of 33 older people who were relocated due to flooding in Depok City ingested the snack bars provided to them. During this particular time frame, it was noted that the average weight of the aforementioned retired individuals rose by 0.2% (6).

The primary objective of this study was to analyze a specific type of edible film-forming product (EFP) in the form of snack bars. The EFP was composed of *api-api* mangrove flour and sword bean. The *api-api* mangrove was chosen for examination due to its few studies compared to the lindur mangrove. Carbohydrates are present in this specific type of mangroves, albeit at a lower concentration (9). The nutritional makeup of 100 g of *api-api* mangrove is as follows: the liquid sample is composed of 21.43 g of carbohydrates, 10.4 g of protein, 0.043 g of fat, and 22.24 mg of vitamin C per milliliter (12). The study conducted by mangrove sword bean food bars reveals that their product has 55 g of carbohydrates, 24 g of protein, and 3 g of fat per 100 g (13). Food bars that utilize *api-api* mangrove flour have the potential to be a feasible and nourishing alternative to wheat flour (7). However, incorporating *api-api* mangrove flour in producing baked goods such as cookies and biscuits is not commonly practiced. The daily caloric needs of older women and men, total 1,900 kcal, were met by consuming four snack bars of a specific type, each weighing 100 g resulting in a total caloric intake of 2,000 kcal.

This research aimed to examine the possible impact of a snack bar made from *api-api* mangrove (*A. marina*) and sword bean (*C. ensiformis*) on the weight and Body Mass Index (BMI) of older people afflicted by landslides. The snack bars can also be used as emergency food in case of various other types of natural disasters such as floods, earthquakes, volcanic eruptions, etc. Several research have been conducted to investigate emergency food formulations in Indonesia. Numerous research investigations have been conducted in Indonesia to investigate emergency food compositions. These investigations had examined the feasibility of utilizing various ingredients, such as white millet and red bean flour, in producing food bars (8). Researchers have also explored the potential of incorporating moringa flour into cookies (14) and investigated the viability of combining sweet potato flour, banana flour, and mung bean flour in emergency food formulations (5). Nevertheless, the combination of *api-api* mangrove flour and *koro pedang* bean flour has yet to be utilized thus far in developing food bars. However, the *api-api* mangrove flour has been recognized as a prospective alternative to rice and wheat flour in manufacturing biscuits. This is attributed to its elevated energy content surpassing rice and maize. The inclusion of *koro* sword nut flour results in an elevation in the levels of lipid content, carbs, protein, zinc, and vitamin C. The inclusion of koro sword nut flour results in an elevation of lipid content, carbs, protein, zinc, and vitamin C levels. The potential impact of snack bars comprising a combination of *api-api* mangrove flour and *koro pedang* bean flour on the nutritional wellbeing of young children and older people affected by landslides remains unexplored in existing research. The potential effectiveness of snack bars is composed of a combination of *api-api*.

## Materials and methods

### Research design

A non-randomized pre-post intervention study was conducted, involving a sample of 31 older individuals who were divided into treatment and control groups. These participants were affected by the landslide catastrophe (15). Ethical approval (No. 089/KEPPKSTIKSC/VII/2022) was obtained by the Health Research and Development Ethics Committee (KEPPK) of Sint Carolus, College of Health Sciences, Jakarta. Before commencing the 2-week study period from August 10 to August 25, 2022. The intervention lasted only 2 weeks because emergency food is provided for refugees or victims of natural disasters for 15 days from the time a disaster is experienced (16). All eligible participants provided their informed consent by signing a formal document at the onset of August 2022. The signing event occurred as part of the introductory activities of the study, and the *posbindu* cadres and the research team witnessed it.

### Subject and population

The study sample comprised older people impacted by the landslide calamity in Cihanjuang Village, Cimanggung Subdistrict, Sumedang District, West Java Province, Indonesia. The geographical location of this village falls within the administrative boundaries of the West Java Province in Indonesia. The present study utilized the following criteria to determine the eligibility of older persons for inclusion: the study's inclusion criteria encompassed individuals aged 60 years or older, of both genders, who lived in Cihanjuang Village, who were directly affected by the landslide incident, and who exhibited normal, under, or over-nutrition statuses. To be eligible for the study, individuals were required to have no pre-existing chronic or degenerative disorders and demonstrate a desire to abstain from snacks other than snack bars and plain water.

The researchers employed the formula for the hypothesis test of paired mean difference (17) to determine the minimum required sample size. The above conclusion was deduced based on an observed augmentation in body weight of 0.2 kg within the older demographic, accompanied by a standard deviation of 0.1 (6). A significance level of 0.05 was selected for conducting bilateral hypothesis testing during this inquiry. Therefore, to attain a statistical power of 90%, it was essential to possess a sample size (*n*) of 15. The nutritional status of all eligible participants, who were older persons, was evaluated based on the established inclusion criteria. An anthropometric assessment was conducted from August 2 to August 6, 2022. These assessments encompassed the measurement of weight and height, as well as the application of the Mini Nutritional Assessment (MNA) tool. Food bars were supplied to all participants for 2 weeks (18), specifically from August 10 to August 25, 2022. The assignment of individuals to treatment and control groups was chosen based on the residential neighborhoods they inhabit.

### Instrument

At the initiation of the inquiry, the investigators assessed the participants' weight, height, and Mid-Upper Arm Circumference (MUAC). A digital scale with a precision of 0.1 kg was utilized for the three weigh-ins at the beginning, week 1, and week 2 of the study. At the onset of the investigation, a solitary height measurement was acquired utilizing a microtoise device possessing a precision level of 0.1 cm. Mid-upper arm circumference (MUAC) was measured at the baseline using a midline tape, with a single measurement being acquired. The researchers assessed the subjects' malnutrition likelihood using the Mini Nutritional Assessment (MNA) (19). The evaluation of an individual's degree of autonomy can be enhanced by employing various instruments, such as the Basic Activity Daily Living (BADL) and Instrument Activity Daily Living (IADL) scales (20). A Mini-Mental State Examination (MMSE) was employed as a standardized screening technique to determine the existence of dementia or possible cognitive decline (21). During the preliminary stage of the research project, data was gathered on the participants' levels of autonomy, vulnerability to malnourishment, and degree of cognitive decline. The research team created instructional resources, including a brochure and flipchart, to disseminate information regarding a well-balanced diet.

### Subject selection

**Figure 1:**
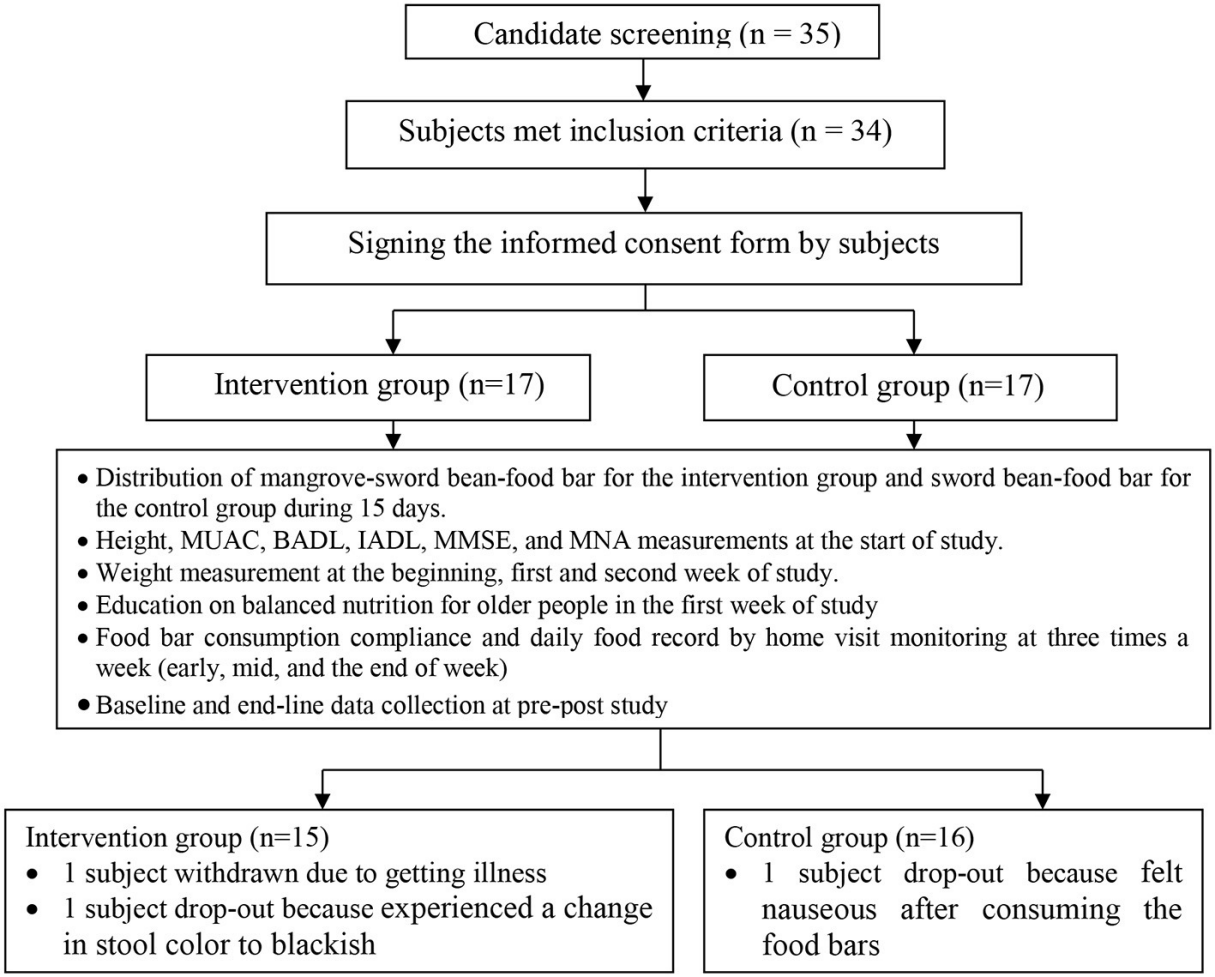
Research scheme of older people.

During the study, a cohort of 31 older people actively participated. The study included 31 senior participants who completed the study, with 16 persons assigned to the control group and 15 individuals assigned to the intervention group with a non-random method. The intervention group comprised of older people from three neighborhoods: 1, 2, and 3. The control group consisted of older people from the neighborhoods 4, 5, and 6. At the initiation of the research endeavor, there existed a total of 34 individuals classified as seniors in each of the two groups who satisfied the stipulated criteria for inclusion in the study. However, three participants of advanced age withdrew from the study due to sore throat disease, a modification in the coloration of their feces to a dark shade, and experiencing nausea after consuming the snack bars (Figure 1)—the aforementioned adverse effects manifested after the ingestion of the snack bars by the individuals. The list of older people who perished in the landslides was obtained from the leader of the integrated health program for older people, *posbindu*. Anthropometric measurements were conducted at various *posbindu* sites to obtain preliminary data regarding the nutritional status of the subjects. The prospective participant was also invited to attend the study's inaugural event 15 days before their intended involvement.

### Nutritional intervention and monitoring

**Figure 2:**
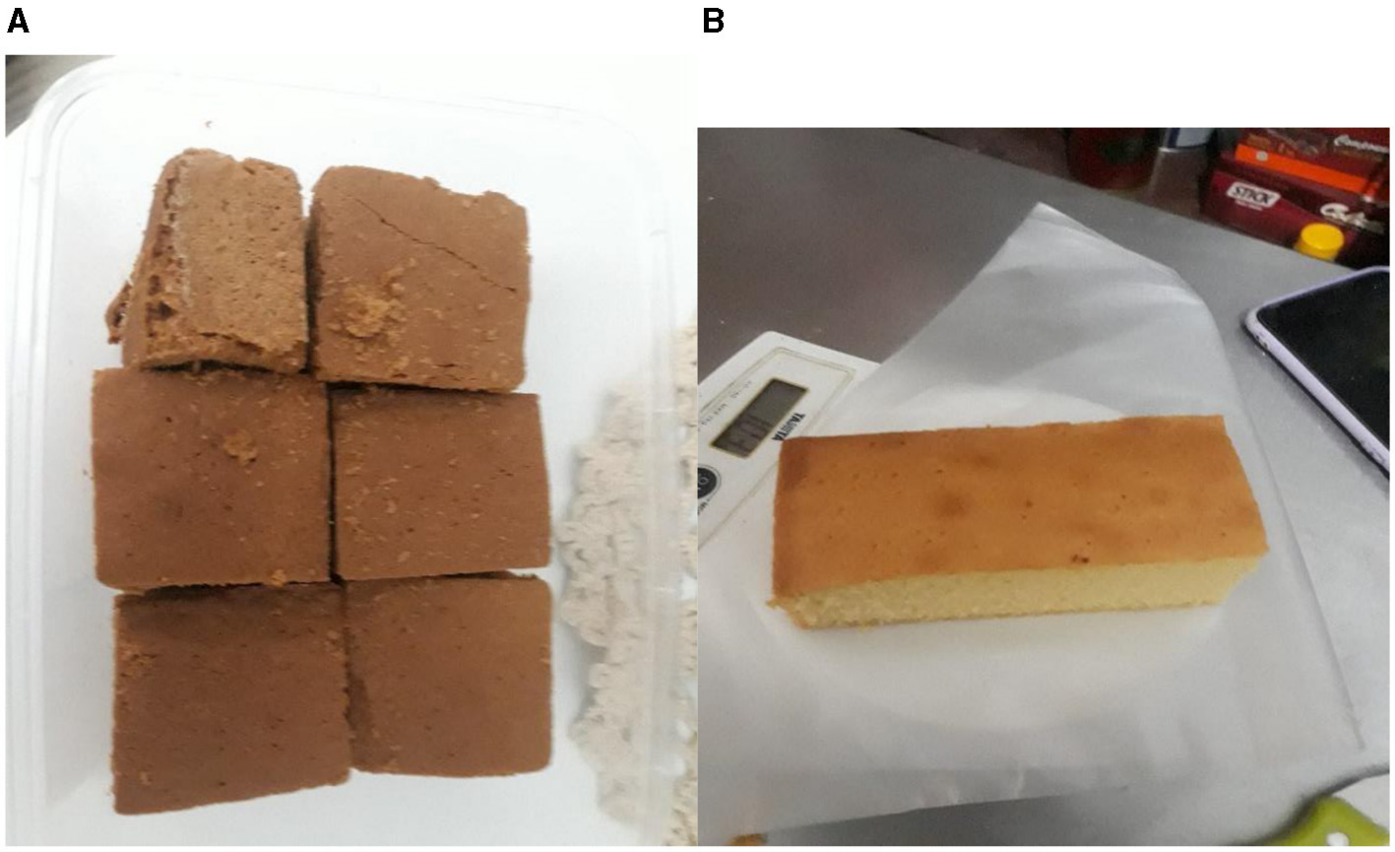
Mangrove sword bean food bar **(A)** and sword bean food bar **(B)**.

For the 15-day study period, individuals assigned to the intervention group consumed mangrove sword bean food bars, whereas those in the control group consumed sword bean food bars (Figure 2). Due to the provision of emergency food assistance to victims of natural disasters and refugees for 15 days following the occurrence (18), the intervention time was limited to 2 weeks. As a component of this experimental study, it is anticipated that each participant within every group will be required to regularly ingest a snack bar with a weight of 50 g. Based on the data presented in Table 1, it can be observed that a 50-g mangrove sword bean snack bar contains a total of 234.2 calories, 24.7 g of carbohydrates, 5.8 g of protein, and 12.5 g of fat. The enumerators used the baseline questionnaire to interview all older people individuals to understand their sociodemographic characteristics comprehensively. These characteristics encompassed age, marital status, educational attainment, occupation, and cohabitation arrangements within their households.

**Table 1:** Nutrient content per 50 g of food bar.

**Type of food bar**	**Energy (kcal)**	**Carbohydrate (g)**	**Protein (g)**	**Fat (g)**
Mangrove-sword bean	234.2	24.7	5.8	12.5
Sword bean	230.4	26.5	3.9	12.1

Nutritional content for results in Table 1 were examined at the Saraswanti Laboratory, Bogor City.

Furthermore, the survey assessed the participant's understanding of balanced nutrition, encompassing topics such as a selection of nutritious foods, the advantages of carbohydrates, proteins, vitamins, and minerals, the definition of healthy food, the concept of balanced nutrition, the importance of weight measurement, the purpose of stable or reduced weight, including its underlying causes and consequences, and strategies aimed at weight gain. The participants in a group received balanced nutrition education twice for 30 min in the first and second weeks of the study. Because practically all of the participants did not understand the Indonesian language, the nutrition education materials that were presented to the Sundanese population by the local enumerators, except the questionnaire, MNA, and MMSE tools.

The data collected at the endpoint of the study were acquired after the administration of instructional interventions to assess the extent to which modifications in balanced nutrition and macronutrient intake had occurred before selecting the mangrove-sword bean-food bar as the Emergency-Friendly Product (EFP) for older people, a hedonic assessment was conducted. The objective of the experiment was to ascertain the relative popularity of different combinations of *api-api* mangrove and sword bean in food bars. To complete the organoleptic test, a cohort of 20 individuals of advanced age, lacking any specialized expertise, was selected from diverse geographical locations and assigned to designated study sites. Ten to 20 people is the ideal number of panelists for a panel that is not informed (21). Due to the inherent possibility of bias, specific panelists with less training declined to participate in the study. The many attributes of the snack bar, such as its color, aroma, flavor, and texture, might impact individuals' compliance with and adoption of the food. The smell, texture, color, and taste of a snack bar were assessed using a four-point hedonic scale, with ratings ranging from 1 (indicating great hate) to 4 (displaying strong liking) (21, 22).

### Monitoring for adherence to consumption regulations in food bars

During the duration of the research, the dietary habits of older people were monitored through regular home visits and anthropometric measures (weight) taken at *posbindu* every week during the study. Weight gain over 2 weeks was used as an indicator of adherence to consuming snack bars. During each household visit, detailed records were documented about the consumption of food items, the allocation of cookies, the remaining quantity of cookies, and the consequences following the consumption of cookies. At the onset, one older people individual expressed experiencing a headache due to consuming cookies, while another older people individual reported experiencing difficulties in bowel movements after consuming cookies. These remarks were made regarding the adverse effects associated with the consumption of cookies. Subsequently, the enumerators proceeded to elucidate to the participants that the detrimental repercussions observed in the initial stages were indicative of an early adaptation.

Furthermore, the adverse effects will diminish after the body has acclimated to the medicine. During food bar distribution, proficient enumerators conducted household visits to collect data on individuals' food intake records. The frequency of these visits was scheduled at a rate of three times each week. Anthropometric data was collected during the initial and subsequent weeks of the study to check compliance with food bar intake among the older people participants. Furthermore, food recalls were documented weekly, namely at intervals of 24 h, 3 days, and 7 days.

### Food bar preparation technique

The food bar known as the *api-api* mangrove sword bean bar is composed of *api-api* mangrove flour and sword bean flour, melted butter, poultry eggs, refined white sugar, a modest quantity of wheat, and chocolate, strawberry or orange paste. The food bar was fabricated employing a digital oven, a mixer, a 20 cm × 15 cm alumunium brownie mold, a digital scale, a food bar dough-cutting equipment, and a dough mill. The following are the prescribed protocols for preparing food bars: the process involves the combination of melted butter and white refined sugar. The dough underwent mixing using a mechanical device for 5 min, resulting in a state of uniformity. Incorporate the *api-api* mangrove flour into the mixture, and afterward, add the sword bean flour, egg yolks, and pasta. Combine the dough with a sizable wooden spatula until it achieves a consistent and homogeneous texture. Transfer the batter onto a rectangular baking dish designed for brownies and cook it in a digitally controlled oven for 30 min, maintaining a temperature of 180°C.

### Data analysis

To analyze the frequency distribution of the food bar organoleptic test, a univariate analysis was conducted using SPSS Version 22. The socio-demographic attributes considered in this analysis included age, gender, marital status, most recent education level, most recent position, living arrangement of family status, nutritional status, and knowledge of balanced nutrition among older people. The MNA, BADL, IADL, MMSE, and MUAC are all administered in a manner that is similar to the previously stated method. The Nutri Survey Program was employed to analyze the data acquired on food consumption for 2 weeks. The data analysis aimed to compare the consumption amounts for the four macronutrients (energy, carbohydrate, fat, and protein) before and after the study using 3 days of 24 food recall (at the first, middle, and end of weeks). Bivariate analysis, and paired *t*-tests were employed to assess the alterations in mean weight, BMI, macronutrient consumption (specifically energy, carbohydrate, protein, and fat), and comprehension of balanced nutrition throughout the study. In all investigations involving two opposing perspectives, a *p*-value of 0.01 was deemed statistically significant.

## Results

The color, aroma, taste, and texture were the four factors that were considered in the study to determine whether the mangrove-sword bean food bar was preferred. In comparison to the sword bean food bar, the mangrove-sword bean food bar produced a significantly higher mean score for color, scent, taste, and texture when evaluated by older individuals (Table 2). Table 3 shows the socio-demographic parameters, nutritional status, level of independence, and cognitive function of older people. The study participants in the intervention group were mostly men, while the women in the control group were mostly married. The intervention group's mean age was slightly older than the control group. Almost all participants in the two groups had a low level of formal education, lived with their children and grandkids, and had retired. The majority of people in both groups had normal nutrition status (62.4% in the control group and 66.7% in the intervention group), normal MUAC (more than 23.0 cm), independence level for basic daily and instrumental activities, and normal cognitive performance. However, there were members in both groups who had cognitive impairment and, most likely, cognitive decline (Table 3).

**Table 2:** Organoleptic test of mangrove-sword bean and sword-bean food bars by older people.

**Parameter**	**Mangrove-sword bean food bar**	**Sword bean food bar**
Color	3.7 ± 0.7 (like)	3.0 ± 0.6 (like)
Aroma	3.5 ± 0.8 (like)	2.2 ± 0.1 (dislike)
Taste	3.5 ± 0.7 (like)	2.0 ± 0.5 (dislike)
Texture	3.0 ± 0.7 (like)	2.1 ± 0.4 (dislike)

1 = very dislike; 2 = dislike; 3 = likes; 4 = very like.

**Table 3:** Socio-demographic characteristics, nutritional status, independence level, and cognitive performance of older people subjects.

**Indicator**	**Control**		**Intervention**		**Total**	
	***n***	**%**	***n***	**%**	***n***	**%**
**Sex**						
Male	7	43.7	8	53.3	15	48.4
Female	9	56.3	7	46.7	16	51.6
**Marital status**						
Married	8	50.0	9	60.0	17	54.8
Widow/widower	8	50.0	6	40.0	14	45.2
**Age (y.o)**						
Mean ± DS	65.3 ± 5.4		70.2 ± 9.2		67.7 ± 7.8	
60–65	10	62.5	8	53.4	18	58.0
66–74	4	25.0	2	13.3	6	19.4
≥75	2	12.5	5	33.3	7	22.6
**Final education level**						
Low	15	93.7	13	86.6	28	90.3
Moderate	1	6.3	2	13.4	3	9.7
**Staying at home**						
Alone	1	6.3	3	20.0	4	12.9
Husband/wife	1	6.3	2	13.3	3	9.7
Spouse/children/grandchild	3	18.7	5	33.3	8	25.8
Children/grandchild	11	68.7	5	33.4	16	51.6
**Working type**						
Laborer	4	25.0	4	26.7	8	25.8
Private	1	6.3	1	6.7	2	6.5
No Job	11	68.7	10	66.6	21	67.7
**Body mass index (BMI) kg/m** ^2^						
Underweight (< 18.5 kg/m^2^)	0	0.0	1	6.7	1	3.2
Normal (18.5–24.9 kg/m^2^)	10	62.4	10	66.7	20	64.5
Overweight (25.0–29.9 kg/m^2^)	3	18.8	3	20.0	6	19.4
Obesity (≥30.0 kg/m^2^)	3	18.8	1	6.7	4	12.9
Mean ± DS	25.2 ± 4.1		22.9 ± 4.5		24.1 ± 4.4	
**Mid upper arm circumference (MUAC)**						
Mean ± DS	26.3 ± 2.8		25.2 ± 3.5		25.8 ± 3.1	
**Independence level**						
**Basic activity daily living (BADL)**						
Independently	15	93.8	15	100.0	30	96.8
Light dependence	1	6.2	0	0.0	1	3.2
**Instrumental activity daily living (IADL)**						
Independently	13	87.5	12	80.0	25	80.6
Need help now and then	3	12.5	3	20.0	6	19.4
Need help all the time	0	0.0	0	0.0	0	0.0
**Mini nutritional assessment (MNA)**						
Normal	16	100.0	14	93.3	30	96.7
At risk malnourished	0	0.0	1	6.7	1	3.3
**Mini mental state examination (MMSE)**						
Normal	8	50.0	10	66.7	18	58.1
Probably cognitive deterioration	5	31.3	3	20.0	8	25.8
Cognitive deterioration	3	18.7	2	13.3	5	16.1

DS, deviation standard.

Body Mass Index (BMI) was used to determine the nutritional status of older people. The intervention group's older participants experienced a slightly larger average weight gain than the control group. During the 15-day intervention, the BMI indicator revealed identical results. Positive significant differences in post-study weight and BMI were identified within the intervention group, but not between groups (Table 4). Table 5 displays the variations in energy, carbohydrate, protein, and fat consumed by older people before and after the study. The mangrove-sword bean food bar consumption was associated with increased macronutrient consumption, specifically carbohydrates, in the intervention group (*p* 0.02). In contrast, the groups had no significant difference regarding the average amounts of energy, protein, and fat. Balanced nutrition knowledge rose after the study (*p* = 0.001), and there were variations between pre-and post-test scores among the older people.

**Table 4:** The mean difference of weight and BMI of older people at pre-post study.

**Indicator**	**Intervention**		**Control**		**Different**	**#*p***
	**Mean**	**DS**	**Mean**	**DS**		
**Weight (kg)**						
At 1st week	57.2	9.5	52.6	9.1	4.6	0.177
At 2nd week	57.4	9.4	52.7	9.1	4.7	0.167
Difference	0.3	0.4	0.2	0.4	0.1	0.505
95% CI of different	(0.0 to 0.5)		(−0.0 to 0.4)			
*p* ^*^	0.03^**^		0.150			
**BMI (kg/m** ^2^ **)**						
At 1st week	25.1	4.1	22.9	4.5	2.2	0.155
At 2nd week	25.2	4.1	22.9	4.5	2.3	0.147
Different	0.1	0.2	0.1	0.1	0.1	0.408
95% CI of different	(0.0 to 0.2)		(−0.0 to 0.2)			
*p* ^*^	0.022^**^		0.152			

^#^*p*, Independen t-test.

^*^p, Dependent t-test.

^**^p < 0.05 at significant level.

CI, confidence interval; DS, deviation standard.

**Table 5:** Comparison of mean macronutrient intake and nutrition knowledge of older people.

**Indicator**	**Intervention**	**Control**	***p***
	**Mean** ±**DS**	**Mean** ±**DS**	
**Energy (kcal)**			
At 1st week	1,173.9 ± 5,20.7	1,162.8 ± 370.1	0.946
At 2nd week	1478.3 ± 629.9	1,403.6 ± 529.3	0.724
*p* ^*^	0.072^**^	0.112	
**Carbohydrate (g)**			
At 1st week	179.2 ± 80.9	188.7 ± 56.7	0.709
At 2nd week	238.9 ± 100.6	229.8 ± 94.5	0.797
*p* ^*^	0.020^**^	0.071	
**Protein (g)**			
At 1st week	38.6 ± 18.5	36.4 ± 14.4	0.728
At 2nd week	41.3 ± 24.1	42.2 ± 16.3	0.905
*p* ^*^	0.645	0.283	
**Fat (g)**			
At 1st week	35.1 ± 22.8	30.5 ± 20.0	0.562
At 2nd week	39.9 ± 23.5	34.9 ± 17.3	0.502
*p* ^*^	0.601	0.503	
Total consumption of food bar (g)	643.8 ± 25	640.0 ± 38.7	0.749
**Balanced nutrition knowledge**			
Pre-study	30.6 ± 16.1	26.6 ± 14.6	< 0.001^**^
Post-study	64.2 ± 14.3	67.2 ± 13.9	< 0.001^**^

^#^p, independent t-test.

^*^p, dependent t-test.

^**^p < 0.05 at significant level.

DS, deviation standard.

## Discussion

The objective of research was to investigate the potential effects of a snack bar composed of api-api mangrove (*A. marina*) and sword bean (*C. ensiformis*) on the weight and BMI of older people affected by landslides. Despite its potential as a suitable alternative to rice and wheat flour, the possibility of utilizing api-api mangrove (*A. marina*) to produce food bars has yet to be previously investigated. According to a study by researchers (7), api-api mangrove has a greater energy density than rice and corn. Older people are prone to moderate to high risk during a crisis due to sensory abnormalities, physical restrictions, and degenerative disorders (23). The likelihood of surviving a natural disaster is influenced by various factors, including physical mobility, hearing impairment, limited physical mobility, poor vision, and memory deficits (24, 25). The issue of insufficient provision of sustenance for individuals affected by disasters should be considered. The nutritional status of individuals may experience deterioration after a natural catastrophe due to many factors, such as reduced access to health services, disruptions in food delivery routes, and insufficient sanitation (26). It is confident that there will be a heightened demand for healthcare and sustenance in regions impacted by disasters. Hence, all relevant parties must prioritize the effective administration of response strategies, particularly those that address the nutritional wellbeing of individuals affected by disasters. The unmet nutritional needs of older people at post-disaster are susceptible to infectious diseases and malnutrition (27). Following a devastating event, the issue of restricted availability of healthcare services and enough nourishment arises. In contrast, older people must have a diet rich in essential nutrients to fulfill their daily nutritional requirements and carry out their daily tasks.

Many individuals exhibit a typical nutritional status at the commencement, while a subset presents with excessive weight or obesity. The average MUAC of both groups exceeded 21 cm, a widely accepted value within the normal range (28). During instances of emergency, such as natural catastrophes, the use of Mid-Upper Arm Circumference (MUAC) proves to be an appropriate screening tool for evaluating the nutritional condition of older people adults. According to the MNA findings, the vast majority of individuals in both groups had a normative nutritional status. MNA has been widely employed as a convenient method for assessing the nutritional status of older people (28, 29).

Upon the study's conclusion, it was observed that the intervention group exhibited a slightly higher degree of weight gain and BMI alteration than the control group. Mangrove sword bean food bars in the diet impacted the development of nutritional indicators due to carbohydrates in these food bars (8, 9). The intervention group exhibited a higher mean consumption of these food bars than the control group. Carbohydrates are classified as a macronutrient that facilitates energy production, leading to an elevation in calorie intake and BMI (30). Approximately 11% of older people can meet their daily carbohydrate requirements by regularly consuming 50 g of mangrove sword bean snack. According to a study (31), the *api-api* mangrove flour has a fat content of 0.04 percent, protein content of 10.4 percent, and carbohydrate content of 21.4 percent per 100 g. The findings of the current study on the weight gain experienced by older people align with those observed in a similar study conducted on older people flood victims in Depok City (6).

According to the study conducted by Fatmah et al. (6), there was a recorded weight gain of 0.2 kg for 15 days following the intervention. The investigation findings are corroborated by a study on food and nutrition assistance for older people after an earthquake disaster (32). Upon the study's conclusion, it was observed that there existed a notable disparity in the level of knowledge of balanced nutrition among the senior participants of each respective group. The study's outcomes suggest that the nutritional knowledge of aged persons was statistically consistent with previous research, which indicated that their overall understanding of a balanced diet improved following nutrition education (33). In the aftermath of a disaster, nutrition management activities are implemented during rebuilding and rehabilitation to enhance and sustain the nutritional wellbeing of those affected by the event (34). The unique nutritional requirements that mean the need for energy decreases, but the need for protein remains of older people in the aftermath of a disaster have not been well addressed.

The provision of nourishing food and nutrition education has the potential to effectively mitigate malnutrition among older people individuals in the aftermath of natural calamities while also alleviating food insecurity. Food insecurity can be alleviated through emergency food assistance programs. The enhancement and preservation of the nutritional and health condition of individuals affected by a disaster can be achieved by implementing nutrition management strategies. These strategies encompass surveillance measures such as anthropometric assessment of older people and nutrition awareness initiatives involving nutrition education and the utilization of snack bars. These activities respond to and build upon the information gathered from public health service endeavors. The ability of individuals and institutions to cultivate a localized safety culture can enhance resilience in the aftermath of a disaster. Maintaining a constant state of readiness involves planning to ensure individuals, families, and communities are prepared for all types of disasters that may impact the community. The steps include preparing for emergencies, creating a disaster plan, and staying informed about potential threats using traditional local wisdom such as poetry, fairy tales, and construction of house on stilts (35). Integrating food security into post-disaster recovery initiatives is crucial to uphold the nutritional and health wellbeing of populations particularly susceptible to the impacts of disasters, such as older people. The Sendai Framework emphasizes the mitigation of catastrophe risk within people of older individuals (36). The post-disaster recovery process is impacted by heightened community engagement by observing emergency food consumption protocols.

## Conclusion

Following a natural disaster, older individuals were provided with a dietary supplement in the form of a snack bar containing mangrove sword beans for 15 days. As a result, these individuals saw a modest increase in body weight, with an average gain of 0.2 kg. Consumption of carbohydrates in mangrove sword bean snack bars directly impacts weight when used as a dietary supplement. While the BMI of older individuals did not exhibit a noteworthy rise, a notable disparity in weight was observed following the consumption of the *api-api* mangrove and sword bean snack bar compared to their pre-bar weight. Due to its nutritional composition and structural attributes, the snack bar remains a viable choice for emergency sustenance options during and following a crisis. Education regarding appropriate nutrition is crucial in disaster relief initiatives, particularly when considering particularly susceptible demographics such as older people and children underfive.

Further investigation utilizing a more expansive cohort and an extended temporal scope is imperative to generate empirical data on the effects of emergency sustenance, such as snack bars, on the nutritional wellbeing of displaced individuals in the aftermath of catastrophic events. This assertion holds particular significance for populations that are considered vulnerable, such as older people, children below the age of five, lactating women, and expectant mothers. The present research endeavors to comprehensively address various nutritional statuses observed in the older people population, including persons who are underweight, of average weight, overweight, and obese. In addition, Law 24 of 2007 on Disaster Management in Indonesia is a suitable legal reference for disaster management in Indonesia. However, it does not adequately protect older people group prone to disasters. Therefore, it is recommended that specific provisions for older people for disaster management be included in the draft of the Older People Law. Government policy to conduct rapid nutrition and health assessments of older people to identify nutritional problems during emergencies and post-disasters.

## Data availability statement

The original contributions presented in the study are included in the article/supplementary material, further inquiries can be directed to the corresponding author.

## Ethics statement

The studies involving humans were approved by the Ethics Commission for Health Research and Development (KEPPK) of the Sint Carolus, College of Health Sciences, Jakarta (No.089/KEPPKSTIKSC/VII/2022). The studies were conducted in accordance with the local legislation and institutional requirements. Written informed consent for participation in this study was provided by the participants' legal guardians/next of kin.

## Author contributions

FF: Conceptualization, Data curation, Formal analysis, Funding acquisition, Investigation, Methodology, Resources, Supervision, Validation, Visualization, Writing – original draft, Writing – review & editing.
